# Multiple Performance Optimization for Microstrip Patch Antenna Improvement

**DOI:** 10.3390/s23094278

**Published:** 2023-04-26

**Authors:** Ja-Hao Chen, Chen-Yang Cheng, Chuan-Min Chien, Chumpol Yuangyai, Ting-Hua Chen, Shuo-Tsung Chen

**Affiliations:** 1 Department of Communication Engineering, Feng Chia University, Taichung 40724, Taiwan; 2 Department of Industrial Engineering and Management, National Taipei University of Technology, Taipei 10608, Taiwan; 3 Department of Industrial Engineering, School of Engineering, King Mongkut’s Institute of Technology Ladkrabang, Bangkok 10520, Thailand; 4 Department of Applied Mathematics, Tunghai University, Taichung 407, Taiwan

**Keywords:** antenna optimization, design of experiment, response surface method

## Abstract

As the Internet of Things (IOT) becomes more widely used in our everyday lives, an increasing number of wireless communication devices are required, meaning that an increasing number of signals are transmitted and received through antennas. Thus, the performance of antennas plays an important role in IOT applications, and increasing the efficiency of antenna design has become a crucial topic. Antenna designers have often optimized antennas by using an EM simulation tool. Although this method is feasible, a great deal of time is often spent on designing the antenna. To improve the efficiency of antenna optimization, this paper proposes a design of experiments (DOE) method for antenna optimization. The antenna length and area in each direction were the experimental parameters, and the response variables were antenna gain and return loss. Response surface methodology was used to obtain optimal parameters for the layout of the antenna. Finally, we utilized antenna simulation software to verify the optimal parameters for antenna optimization, showing how the DOE method can increase the efficiency of antenna optimization. The antenna optimized by DOE was implemented, and its measured results show that the antenna gain and return loss were 2.65 dBi and 11.2 dB, respectively.

## 1. Introduction

In IOT applications, many wireless communication modules are used. The antenna is a wireless communication module and an essential circuit component. Antenna characteristics are directly related to circuit applications and application scenarios, and therefore, antennas often must be customized. Consequently, antenna design efficiency has a direct relationship with the time to market of a product and thus is highly influential in product development.

To increase the efficiency of antenna design, extensive research has been conducted on antenna synthesis and design optimization. GA and the method of moments were used to design a broadband patch antenna, increasing the antenna by 20% [1]. In another study [2], the plate geometry signal feed position and the shorting pin position of an inverted F antenna were designed using a GA to optimize the antenna bandwidth and gain. Other researchers [3] have designed an ultra-wideband (UWB) slot antenna by using PSO combined with the neighborhood redispatch technique. In addition to increasing the antenna bandwidth, covering the frequency bands of UWB (3.1–10.6 GHz) and Bluetooth (2.4–2.484 GHz) with the 5.15–5.825 GHz cut-off band, a shallow opportunity to avoid signal interference with each other occurs. A GA–SA approach was used to improve the voltage standing wave ratio of a high-frequency antenna, and the transducer power gain was also improved to enhance gain performance [3]. Related evolutionary algorithms have also been proposed [4,5,6,7,8]. The IWO algorithm was used to realize a flat antenna with a U-hole, and thus the target antenna characteristics were achieved [9].

Chen and Ku [9] stated that the traditional process of antenna optimization is based on a heuristic algorithm. However, because it uses a blind search, it is very time-consuming. To address this, they used full factorial design and orthogonal fractional experiments to design the antenna. Furthermore, they conducted three experiments that showed that the orthogonal design method provides the advantages of efficiency and accuracy, and can shorten the design cycle. Chen [10] designed a UWB antenna by using a multi-objective fractional factorial design (MO–FFD). The results showed that the average fidelity factor of E and H planes were 0.88 and 0.84, respectively, indicating a strong correlation between the receiving and emission signals of the antenna. This confirmed that MO–FFD is particularly suitable for UWB antenna design. Dengiz and Belgin [11] used simulation optimization along with response surface methodology (RSM), DOE, simulation modeling, and sensitivity analysis and found that the simulation model could then be analyzed more efficiently.

In this study, a design of experiments (DOE) method is proposed to improve the efficiency of antenna optimization. Some layout parameters of the patch antenna are analyzed with RSM to optimize antenna gain and input return loss performances. The results show that the estimation of antenna performance with DOE to optimize an antenna is more efficient than optimization with the EM simulation tool. Performance of the antenna optimized by DOE was evaluated, and the measured results show that the antenna gain and return loss were 2.65 dBi and 11.2 dB, respectively.

## 2. Materials and Methods

This research focused on the gain of a patch antenna. The aim was to shorten the time spent developing the antenna. We used the experimental design method to identify crucial factors and determine the scope of their influence, and thus find the optimal factor configuration and gain value so that the reflection loss is less than −10 dB. We then used antenna simulation software to verify the results. Traditionally, antenna design has involved using computer-aided engineering simulation software and the finite element method to simulate the electromagnetic field, and the characteristics of the radiation field of the designed antenna are then obtained. However, this method is excessively time-consuming, reducing the efficiency of antenna design. To shorten the development time and increase efficiency, this study (i) used a systematic experimental design method to determine the important factors and the scope of their influence, in addition to performing factor programming experiments, (ii) used RSM to determine the optimal factor combination, and (iii) employed antenna simulation software to verify that the identified combination of factors is optimal.

### 2.1. Antenna Structure

In an antenna, radio waves begin at the internal feed. They then pass through the conductor between the chip and ground excitation from the radio frequency electromagnetic field, and then proceed through the patch around the ground surface and the gap to the outside. The size of the conductor and ground surface can be adjusted to achieve the target resonant frequency band.

**Figure 1:**
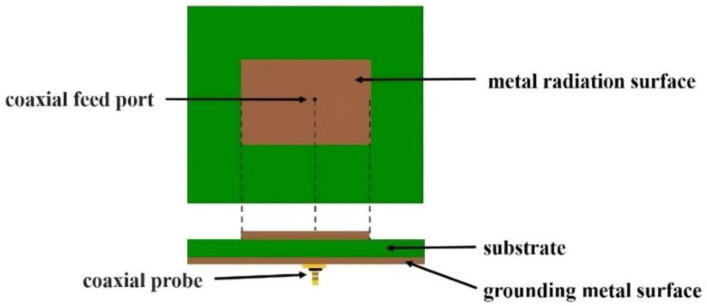
Basic structure of the rectangular microstrip antenna.

A microstrip antenna is situated in a thin substrate with a thin layer of metal as a ground surface. Photolithography corrosion can be used to shape the metal patch, which acts as a radiating surface, and then a microstrip line with a coaxial probe is used for the patch feed. Microstrip antennas can be circular, rectangular, or ring-shaped. This study used a rectangular microstrip antenna, with the following characteristics (Figure 1):

1. Rectangular metal radiation surface: located in the top layer in the middle of the rectangle, it sends and receives signals.
2. Insulating substrate: located in the middle layer, it consists of insulation material with a dielectric constant of 4.4 (e.g., FR4 glass fiber substrate).
3. Ground metal surface: located in the bottom layer; for copper, the area must be larger than the rectangular metal surface.
4. Coaxial probe: a small round coaxial conductor, where the metal surface is connected to the ground and the internal surface is connected to the metal surface. This feed is within the volume of the feed surface, and the energy through the feed surface flows into the antenna.
5. Shorting pins: usually connected to two media, they may gain but contribute to an increase in bandwidth.

### 2.2. Antenna Design Parameters

Two antenna design parameters we used as the design parameters for optimization of antenna gain and reflection loss.

1. Antenna gain can be used to measure the antenna’s directivity, which refers to the capacity of the antenna to transmit signals in a specified direction. Usually, the greater the effective area of the antenna is, the higher is the gain. Gain is measured in dBi units.
2. Reflection loss concerns the feedback component of the feedback signal. The antenna reflection loss should be as small as possible, and at least −10 db. The smaller the reflection loss is, the greater is the signal input, and thus, the greater the radiation power.

### 2.3. Antenna Design Simulation Software

The Advanced Design System (ADS; Keysight, Wokingham, UK) was used for antenna design simulation [12]. Keysight developed microwave circuit and communication system simulation software primarily for RF, microwave, and high-speed digital applications, including SPICE-like simulation, harmonic balance, linear analysis, communication system simulation, and EM simulation. Designers can use this system for analog, RF, and microwave circuit and communication system design simulation analysis.

### 2.4. Design of Experiments

Minitab 18 statistical software was used to execute the experimental design using RSM. RSM supplements the prediction model by combining mathematical and statistical methods so that the experimental area can be adjusted according to the experimental scope to determine the ideal response value.

First, we determine the variables that are influential to the system, and then we select an appropriate level as the initial value of the experiment. A two-level factorial experiment is performed initially. Many parameters often must be considered in the design of an antenna, and the responses are usually affected by the main effect and low-order interaction. A two-factor interaction is therefore appropriate for the model [9]. The formula is as follows:

(1) $$\;\hat{Y}={\hat{\beta }}_{0}+\overset{k}{\underset{i=1}{\sum }}{\hat{\beta }}_{i}x_{i}+\overset{}{\underset{}{\sum }}\overset{}{\underset{i<j}{\sum }}{\hat{\beta }}_{ij}x_{i}x_{j}$$

Where $\hat{Y}$ is an estimated response, which is a function of $x_{1},\ldots ,\;x_{k}$ variables. ${\hat{\beta }}_{0}$ is the intercept. ${\hat{\beta }}_{i}$ and $\;{\hat{\beta }}_{ij}$ are regression coefficients. To verify that the model is appropriate, regression analysis must be performed to examine the experimental results and ensure that the regression model is appropriate. The coefficient of a linear regression between the parameter and the response value is obtained according to the judgment coefficient, and the main effect and the interaction plot generated on the basis of the factor experiment are then added. The values closest to the best solution can then be found and used as the basis for the next experiment. Finally, we consider whether the range of factors should be reduced and then design the second experiment.

If the first-order regression model is appropriate, the steepest ascent method is used to search an area where the optimal response value should be located. The purpose of this method is to search for the best point.

At this point, the curvature of the real response surface increases, which means that the first-order model of the best solution can no longer be applied and the second-order model must be used. In general, the central composite design (CCD) is employed as the second-order model because it is very efficient.

For the CCD, a k-factor experiment with a $2^{k}$ factorial design and nc center point were used as an example [13]. After an experiment is conducted with the CCD and the response value is obtained, the second-order regression model must be further tested using the lack-of-fit test. If there is no clear evidence that the model is not appropriate, the second-order regression model is accepted and is given by Formula (2):

(2) $$\hat{Y}={\hat{\beta }}_{0}+\overset{k}{\underset{i=1}{\sum }}{\hat{\beta }}_{i}x_{i}+\overset{k}{\underset{i=1}{\sum }}{\hat{\beta }}_{ii}x_{i}^{2}+\overset{}{\underset{}{\sum }}\overset{}{\underset{i<j}{\sum }}{\hat{\beta }}_{ij}x_{i}x_{j}\;$$

After accepting the second-order regression model, we can begin the analysis using RSM. The second-order function can be represented by the matrix shown in Formula (3) to identify the stationary point. The stationary point may be the maximum, minimum, or saddle point. According to the extreme theorem, if the solution of function (4) at the extreme value of the differential is 0, we can obtain Formula (5). If the characteristic root is negative, the stationary point has the maximum value; if it is positive, the stationary point has the minimum value; otherwise, the stationary point is the saddle point:

(3) $$\hat{Y}=b_{0}+x^{\prime }b+x^{\prime }\hat{B}x$$

(4) $$\begin{array}{c} \mathrm{among}\;x=\left[\begin{array}{c} x_{1}\\ x_{2}\\ \begin{array}{c} .\\ x_{k} \end{array} \end{array}\right],\;b=\left[\begin{array}{c} b_{1}\\ b_{2}\\ \begin{array}{c} .\\ b_{k} \end{array} \end{array}\right],\\ B=\left[\begin{array}{cccc} b_{11} & b_{12}/2 & . & b_{1k}/2\\ . & b_{22} & . & b_{2k}/2\\ . & . & . & .\\ sym. & . & . & b_{kk} \end{array}\right] \end{array}$$

(5) $$\frac{\partial \hat{Y}}{\partial x}=b+2\hat{B}x=0$$

### 2.5. Optimizing Multiple Response Values

RSM often involves multiple response values; therefore, the desirability function can be used to obtain multiple response optimizations. The desirability function can identify the best objective function of a single response value through mathematical transformation based on the upper and lower bounds of the response variable and its target value [14]. Each response value *y_i_* is converted to the desirability function di, where 0 ≤ *d_i_* ≤ 1. When the response value *y_i_* reaches the target, di is equal to 1. If it exceeds the acceptable area, then *d_i_* is equal to 0. The overall desirability function is *D* = (*d*_1_, *d*_2_, … *d_m_*)^1*/m*^, where *m* is the number of responses, *y_i_*.

The next section describes the application of the antenna design. Factor experiments were performed to conduct the initial fitting, and RSM was employed to identify the stationary point. Finally, the response value was optimized to find the optimal combination of parameters.

## 3. Experimental Results

**Figure 2:**
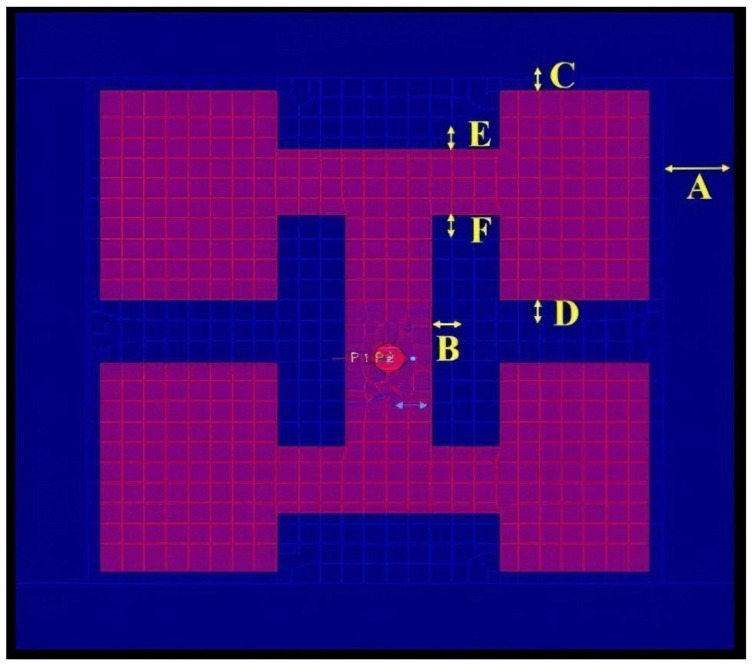
Antenna schematic plot.

The shape of the antenna is shown in Figure 2. The blue area depicts the ground, and the pink area depicts the rectangular metal radiating surface. After the factors of antenna design were screened, six factors, A to F, were selected as the antenna design parameters. The two response variables, i.e., antenna gain and return loss, are shown as gain and RL. Return loss is a measure of the power reflected by an antenna at its input port. It is expressed in decibels (dB) and is defined as the ratio of the power of the incident signal to the power of the reflected signal. A two-level factional factorial design was then used to fit the model. In this research, the objectives were maximum antenna gain and a return loss of less than −10 dB. The optimal experimental combination had to fit these conditions.

### 3.1. Preliminary Experimental Design

The first experimental measurement was based on the current size of the antenna, and we defined the maximum range within which the factors can increase or decrease as the upper and lower limits of two levels. These are shown in Table 1. A fractional factorial design was used to start the preliminary experiments, of which 32 (2^6-1^) were performed.

Minitab 18 statistical software was used to conduct the regression analysis, in conjunction with backward elimination. First, all antenna design parameters were entered into the regression model. The smallest explanatory antenna design parameters were then excluded until all significant changes were removed.

In the preliminary experiments on the gain model, factor A was deleted from the model, indicating that this was not a significant factor in terms of gain. Additionally, we tested the RL model. No factor was deleted; therefore, every factor was significant in this regression model.

To find the optimal combination of antenna design parameters, main-effect graphs were drawn. The main effects of the six factors on gain were identified. Factor A was not significant; factor B, D, E, and F were the smaller the better, and factor C was the larger the better. Regarding the main effects on RL, factors B, D, and E were the larger the better, whereas factors C and F exhibited no significant effects.

The preceding analysis indicates that factors B, D, and E exhibited opposite trends with regard to gain and RL responses. Factor C was the larger the better for gain, but for RL, it exhibited no significant trend. Conversely, factor F was the smaller the better for gain, but for RL, it exhibited no significant trend. These results were employed as benchmarks that enabled us to adjust the factorial range in subsequent experiments.

### 3.2. Central Composite Design

Because the preliminary experiment was not ideal, we adjusted several factors and their ranges for the second trial, as shown in Table 2. The CCD was employed in the original factorial experiment coupled with center points and axial points. Twenty-three experiments were performed and eight axial points were tested; thus, a total of thirty-one experiments were performed for second-order model fitting.

We used background elimination to conduct the regression analysis. We found that all factors were significant in the model for gain. The R-sq was 66%, indicating that the model was appropriate. The formula is presented as follows:

gain = 9.827 + 0.244 A + 0.117 B + 0.015 D – 0.098 E + 0.1469 A × D – 0.0765 A × E + 0.1932 B × D + 0.079 D × E – 0.1224 A × D × E(6)

All factors were significant in the model of RL. The R-sq was 93.21%, indicating that the model was appropriate. The formula is presented as follows:

RL = −14.96 + 2.203 A + 2.837 B − 1.087 D + 5.766 E − 0.607 A^2^ + 0.661 A × B + 0.248 A × E − 0.585 B × D − 1.533 B × E + 0.889 D × E − 0.306 A × B × E(7)

### 3.3. The Optimal Solution of the Response Surface

The objective of this study was to maximize gain and reduce RL to less than −10 dB. Therefore, in the response optimizer in MINITAB, the target of gain was set as the maximum, and the target of RL was set as the minimum. The target was to achieve maximum gain with an RL less than −10. Using Minitab 18 statistical software, we found that the overall desirability function of the prediction model was 0.9271, the desirability function for gain was 0.87864, and that for RL was 0.97823. According to the model, the optimal response values are a gain of 10.6203 and an RL of −27.0495. In these conditions, factor A is 4.5, factor B is −1.5, factor D is −0.4, factor E is −0.1, factor C is fixed at 0.9, and factor F is fixed at −2.4.

Minitab18 was then used to generate a set of feasible solutions in order to determine the values for practical needs. Antenna simulation software was used to verify this set of data, and the results in Table 3 show that for an antenna with a 5.8 GHz input, the return loss is −10.5 dB, and the gain is 9.8. These values are in line with the results from our model, which verifies that the model is applicable.

**Figure 3:**
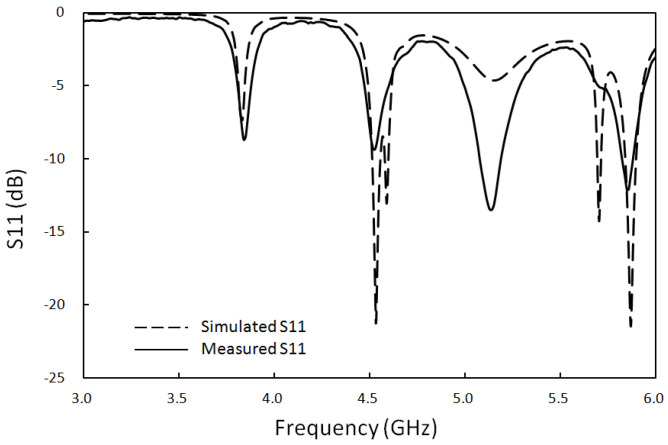
Return loss of the optimized antenna.

**Figure 4:**
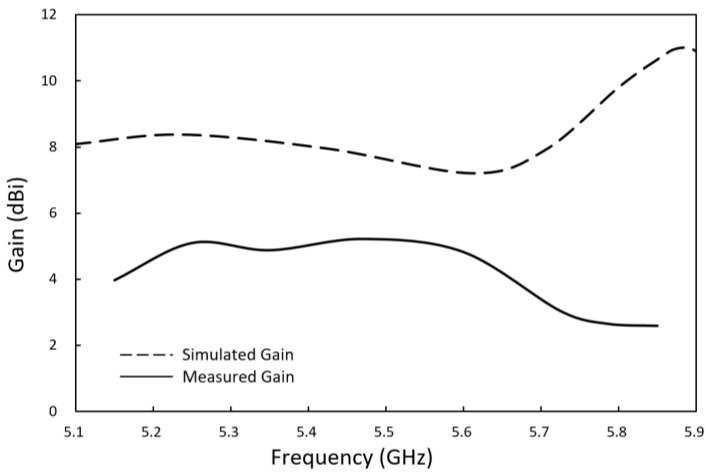
Gain of the optimized antenna.

Using the ADS electromagnetic field simulation software for analysis, the antenna reflection loss and gain characteristics were obtained. As shown by the dotted lines in Figure 3 and Figure 4, the simulated reflection coefficient is 10.4 dB, and the simulated analog gain is 9.8 dBi for an antenna with 5.8 GHz input. The optimal antenna was then used for fabrication on an FR4 substrate.

### 3.4. Verifying the Results

**Figure 5:**
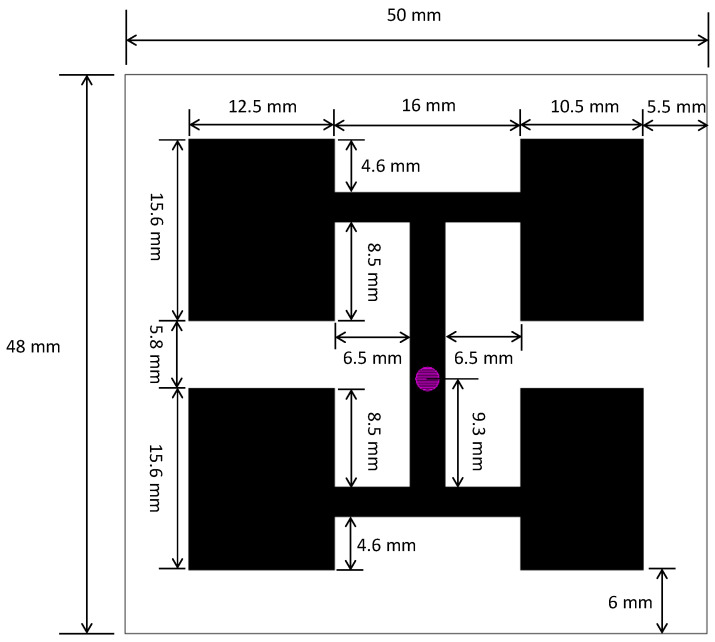
Optimized antenna size.

**Figure 6:**
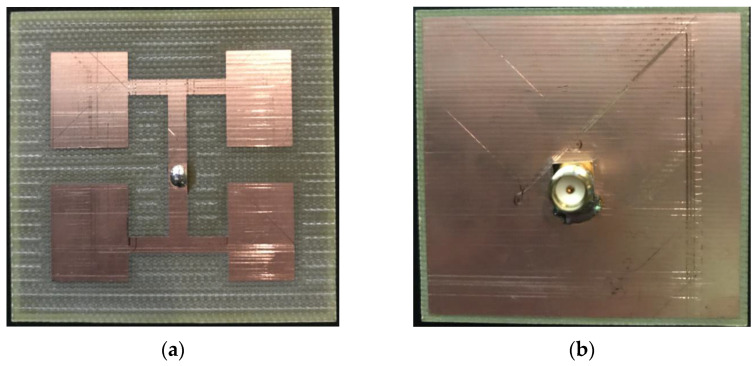
Photo of the physical antenna: (**a**) front and (**b**) back.

Based on the optimized design of the antenna structure shown in Figure 5, the antenna was fabricated on a glass-fiber epoxy substrate (FR4), as shown in Figure 6. In this figure, (a) shows the front of the antenna and (b) the back of the antenna. An SMA connector was used to connect the signal feed point and a network analyzer with a high-frequency coaxial line. The input reflection coefficient S11 of the antenna was measured using the Agilent N5230A network analyzer. Figure 4 shows that the reflection coefficient measured 11.2 dB for an antenna with a 5.8 GHz input. This measurement is very close to the results of the simulation.

**Figure 7:**
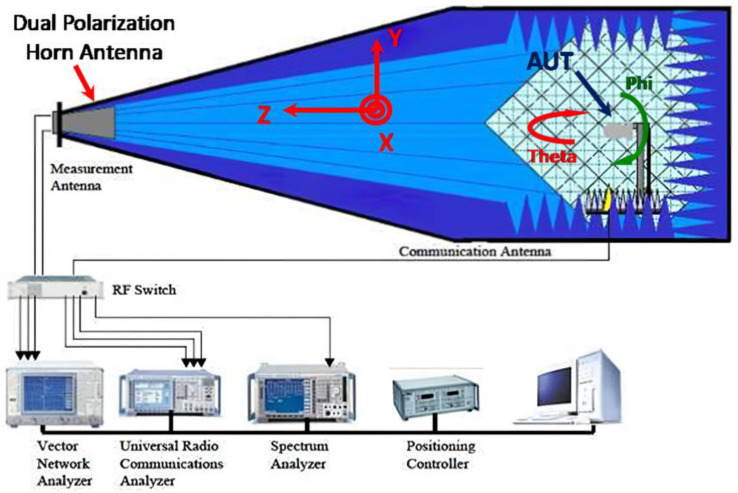
AMS-8600 antenna measurement system architecture.

The AMS-8600 antenna measurement system was used to measure the gain performance of the antenna, Figure 7 depicts the antenna measurement system architecture. The measurement results are shown in Figure 5. The antenna gain at 5.8 GHz was 2.65 dBi, which is somewhat different from the simulation results because the connection between the SMA connector and the antenna caused discontinuities in the signal and the circuit board, leading to parasitic effects. Consequently, the antenna current distribution differed between the simulation and measurement.

### 3.5. Discussion

The findings of this study show how the experimental design method works to increase the effectiveness of antenna design. Using Minitab 18 statistical software, the ideal characteristics of the antenna were identified by setting the target of gain as the maximum and the target of reflection loss as the minimum. The results were then verified using software for antenna simulation, and the fabrication and measurement of the antenna provided additional confirmation. The simulation results and the measured reflection coefficient and gain agreed very closely, proving the accuracy of the optimization technique. Even though parasitic effects caused the measured gain to deviate slightly from the simulation, the results still offer important information about the antenna’s functionality. Overall, the method described in this study can be used to design different kinds of antennas, offering a useful and effective way to maximize antenna performance.

## 4. Conclusions

In this paper, we propose a method for improving the efficiency of antenna design. Using the experimental design method, which involved setting the size of the antenna and the antenna design parameters, we quickly determined the optimal characteristics of the antenna, and then demonstrated the feasibility of this method using simulation software. In this research, an E-type plate antenna was used as an example, and the antenna was designed at 5.8 GHz. The measured reflection coefficient was 11.2 dB and the measured gain was 2.65 dBi. This method can therefore be applied to antenna design to improve efficiency.

## Figures and Tables

**Table 1:** Factors and levels in preliminary trial.

		Factors					
Unit: (mm)		A	B	C	D	E	F
Levels	−1	0	−1.6	−2.2	−3.4	−2.4	−2.4
	+1	5.5	4.9	0.9	2.4	2.2	3.4

**Table 2:** Factors and levels in second trial.

		Factors					
Unit: (mm)		A	B	C	D	E	F
Levels	−1	0	−1.6	Fixed at 0.9	−3.4	−0.1	Fixed at −3.4
	+1	5.5	1.7		−0.4	2.2	

**Table 3:** Predicted and observed data.

		Prediction	Observation
Factor	A	4.5	4.5
	B	−1.5	−1.5
	C	Decided by engineer	0.9
	D	−0.4	−0.4
	E	−0.1	−0.1
	F	Decided by engineer	−2.4
Response	gain	10.62	9.8
	RL	−27.05	−10.4

## Data Availability

The data are not publicly available due to privacy restriction.

## References

[1] Johnson J.M., Rahmat-Samii Y. (1999). Genetic algorithms and method of moments (GA/MOM) for the design of integrated antennas. IEEE Trans. Antennas Propag..

[2] Jayasinghe J., Uduwawala D. (2015). A novel multiband miniature planar inverted F antenna design for bluetooth and WLAN applications. Int. J. Antennas Propag..

[3] Li Y.-L., Shao W., You L., Wang B.-Z. (2013). An improved PSO algorithm and its application to UWB antenna design. IEEE Antennas Wirel. Propag. Lett..

[4] Karimkashi S., Kishk A.A. (2010). Invasive weed optimization and its features in electromagnetics. IEEE Trans. Antennas Propag..

[5] Boudjerda M., Reddaf A., Kacha A., Hamdi-Cherif K., Alharbi T.E., Alzaidi M.S., Alsharef M., Ghoneim S.S. (2022). Design and optimization of miniaturized microstrip patch antennas using a genetic algorithm. Electronics.

[6] Mishra R.G., Mishra R., Chaurasia R.K., Kumari N.P., Kuchhal P. (2022). Particle Swarm Optimization (PSO) for Multi-Objective Performance Optimization of Microstrip Patch Antenna for Wide-band Applications. Proceedings of the 2022 7th International Conference on Communication and Electronics Systems (ICCES).

[7] Pietrenko-Dabrowska A., Koziel S., Mahrokh M. (2022). Optimization-Based High-Frequency Circuit Miniaturization through Implicit and Explicit Constraint Handling: Recent Advances. Energies.

[8] Mishra R.G., Chaurasia R.K., Mishra R., Kumari N.P., Kuchhal P. (2022). Grey Wolf Optimization (GWO) for Multi-Objective Performance Optimization of Microstrip Patch Antenna for Wide-band Applications. Proceedings of the 2022 Third International Conference on Intelligent Computing Instrumentation and Control Technologies (ICICICT).

[9] Chen Y.-S., Ku T.-Y. (2015). Efficiency improvements of antenna optimization using orthogonal fractional experiments. Int. J. Antennas Propag..

[10] Chen Y.-S. (2015). Application of multi-objective fractional factorial design for ultra-wideband antennas with uniform gain and high fidelity. IET Microw. Antennas Propag..

[11] Dengiz B., Belgin O. (2014). Simulation optimization of a multi-stage multi-product paint shop line with Response Surface Methodology. Simulation.

[12] Keysight (Agilent) Advanced Design System (ADS). http://www.keysight.com/zh-TW/pc-1297113/advanced-design-system-ads?cc=TW&lc=cht.

[13] Montgomery D.C. (2014). Textbook: Design and Analysis of Experiments.

[14] Derringer G. (1980). Simultaneous optimization of several response variables. J. Qual. Technol..

